# COVID-19 online teaching intervention and learning performance of college foreign language students

**DOI:** 10.3389/fpsyg.2022.1109032

**Published:** 2023-01-10

**Authors:** Yufeng Xu, Yanfen Zou

**Affiliations:** ^1^Institute of Foreign Languages, Jiangxi Science and Technology Normal University, Nanchang, Jiangxi Province, China; ^2^School of Business Administration, Jiangxi University of Finance and Economics, Nanchang, Jiangxi Province, China

**Keywords:** COVID-19, online teaching intervention, learning performance of college foreign language, DID (difference-in-difference) model, quasi-natural experiment

## Abstract

This quasi-natural experimental study examined an online teaching intervention implemented in response to COVID-19 in China in 2020. It applied the difference-in-difference model to examine the impact and path of the intervention on students’ learning performance of a college foreign language (LPCFL). Based on data from records of withdrawing and changing courses, classroom learning, and teaching evaluations; a questionnaire survey of teachers and students; and relevant school documents during the last seven terms, the results indicated that the online teaching intervention could significantly improve students’ LPCFL. This finding remained robust after adopting a placebo test approach to mitigate possible endogeneity issues. Additionally, this study also conducted a group test through sub-sample regression based on students’ discipline characteristics and intervention organization methods. The results showed that the students who participated in the intervention significantly improved in the three disciplines: humanities was most significantly affected, science and engineering were least significantly affected, and economics and management were in the middle. A range effect was observed for organizational methods. The two downward transmission methods by college teaching management terms had significant positive effects, whereas the other two methods of downward transmission by college student management had significant negative effects. An analysis of the action mechanism indicated that the online teaching intervention mostly improved LPCFL through two channels: students’ learning input and learning support. Overall, these findings not only help expand the research framework on macro environmental intervention policy and micro-learning behavior but also have implications for the in-depth understanding of the real learning effect of online learning interventions for college students and their design in the post-COVID-19 era.

## 1. Introduction

In 2020, the COVID-19 outbreak spread globally, and students’ learning was affected in various countries. This situation promoted the comprehensive transition from traditional to online teaching modes through mandatory institutional change. Previously an exploratory option, online teaching became the only method available, and the physical classroom was transferred to cloud-based live broadcasts. Thus, teachers were required to adjust their traditional teaching designs, while students needed to adapt to new classroom participation methods to cope with the impact of COVID-19 on normal college operations (Bao et al., 2021). Since the 1990s, extensive access to the Internet has greatly promoted the development of online education (Abouhashem et al., 2021). Here, massive open online courses, in particular, experienced explosive growth after 2013. Before the COVID-19 outbreak, a wide range of learners already benefited from online teaching, and large-scale online teaching gradually became a good supplement to traditional face-to-face methods. However, in universities, especially first-class universities, online teaching requires improvements to become the mainstream teaching modality (Qiao et al., 2021). In the nearly 3 years since the sudden outbreak of COVID-19, large-scale online teaching in colleges and universities is no longer a choice but an inevitable trend (Dhawan, 2020), with a shift from simple offline teaching to “large-scale, long-term” online teaching (Mamluah and Maulidi, 2021).

In early 2020, COVID-19 continued to spread in Wuhan, China. On January 27, the Ministry of Education issued a notice on its official website regarding the postponement of the spring semester, requiring universities to adopt the method of “no return to school, no stop to teaching, no stop to learning.” Offline learning activities were suspended in many places, and students were provided with learning support through the Internet and informative educational resources while participating in online teaching and learning activities (Almaiah et al., 2020; Yen, 2020). For college students, by February, 2020, the Ministry of Education had organized 22 online course platforms and opened more than 24,000 free online courses, covering 12 undergraduate disciplines, with many courses offering college credit. These unified action instructions resulted in teaching being transformed from offline to online for approximately 270 million students in China. Facing the arrival of large-scale online teaching, teaching quality and learning effect must be ensured (Zou et al., 2021). Especially in higher education, the development of modern information technology promotes learning to eliminate the limitations of region and time, promote the individual diversity of learning process and learning methods, and strengthen the availability of learning opportunities (Childers and Berner, 2000). However, concerns regarding the impact on quality and effectiveness reflected in factors, such as rates of student retention, course qualification, and degrees awarded have prevented online teaching from being fully integrated into the teaching operations of colleges and universities (Daniel, 2012), despite vigorous promotion from the government and institutional organizations. Further, the uncertainty of the effectiveness of large-scale online teaching makes this problem more prominent. Therefore, it is necessary for us to evaluate students’ online learning intervention to promote the evaluation.

In the context of global integration, foreign languages have become crucial for worldwide communication (Roehr-Brackin and Tellier, 2018). Several countries have developed foreign language courses at the basic education stage and regard foreign language education as an important aspect of national education quality. With the proceeding of China’s reform and opening-up, foreign language learning has received increased attention (Tao et al., 2020). In higher education in China, foreign language courses are listed as public basic courses and have even become important criteria for college graduates. Moreover, popularizing foreign languages, cultivating foreign language talent, and improving foreign language teaching methods and levels are no longer only general teaching problems but also major problems affecting the improved implementation of China’s opening up policy and economic and social development (Zhu et al., 2022). Learning a foreign language has also gained increasing attention from college students and has even become deeply rooted in their minds (Hu, 2018). Therefore, for Chinese college students at this stage, learning foreign languages is an important, continuous and popular course that they started from childhood (Asif et al., 2018; Guo and Qiu, 2018). In particular, following the COVID-19 outbreak, higher education in China has achieved an online teaching practice of “all regions, all coverage, and all directions” (Li and Zhu, 2020). The effects of college students learning foreign languages online and the related influencing factors have drawn the attention of schools and teachers (Li, 2022; Okyar, 2022). This is especially true for English as the most important part of foreign language learning and has always been the most concerned among the most common information. English has become the most widely used language across all aspects of human life (Asif et al., 2018; Guo and Qiu, 2018). According to the announcement of the Ministry of Education in 2020, the proportion of Chinese college students learning English is 91%, which exceeds the popularity of any other course at this stage. Therefore, this study takes English learning as the research object, which has good universality.

Following the COVID-19 outbreak, many scholars have studied the changes of students’ learning efficiency, learning satisfaction, and other aspects in this situation (Almaiah et al., 2020; Yen, 2020), and have shown that many students face wide-ranging challenges in learning autonomy and control (Syah, 2020; Windhiyana, 2020; Teng and Wu, 2021) when taking intensive online courses for the first time (Dhawan, 2020). This can also trigger various negative emotions in students, such as confusion, anxiety, depression, and even weariness and burnout, making it difficult to ensure the effectiveness of foreign language learning (Wu and Li, 2020; Wang and Zhang, 2021; Wang and Su, 2022). Further, large-scale surveys on online teaching during COVID-19 revealed certain problems that largely restrict the effectiveness of online foreign language learning (Boonchuayrod and Getkham, 2019), such as poor communication between teachers and students, students’ weak autonomous learning ability, insufficient student participation (Kahu and Nelson, 2018; Chen and Cao, 2020; Yang et al., 2020), and students falling into the virtual absence state of “online but not studying” (Zeynali et al., 2019). Therefore, many scholars have discussed how to improve foreign language learning (Guo et al., 2022), and posited that learning motivation (Ramkissoon et al., 2020; Purwaamijaya et al., 2021; Pan et al., 2022), willingness (Menéndez et al., 2018; Widayanti and Suarnajaya, 2021), and learning input (Habok and Magyar, 2018; Hou, 2018; Huang et al., 2018) are key factors in facilitating college students’ foreign language learning. Some scholars have argued that the online technology and teaching methods teachers use, as well as their interactions with students, are important factors affecting foreign language learning for college students (Çankaya, 2018; Martin et al., 2019; Cai, 2021). In addition, some scholars believe that online interactive teaching, curriculum structure debugging, learning support, and learning environment are key factors affecting online foreign language learning (Bao, 2014; Bao et al., 2021).

However, previous researches have only analyzed the factors influencing students’ learning performance of college foreign languages (LPCFL), failing to examine the specific effects of either an organized online teaching intervention on LPCFL or the endogeneity between factors during the COVID-19 period. Here, an organizational intervention is a project carried out when universities adopt large-scale online teaching modalities. Therefore, according to data availability, this study used online English teaching at a college in Jiangxi Province (middle of China) for the research sample, and divided students into a treatment group (intervention students) and a control group (non-intervention students) according to whether they participated in the school’s online teaching intervention. The formal implementation of online teaching in response to COVID-19 in the spring semester of 2020 was taken as the landmark event for a quasi-natural experiment. This study applied the difference-in-difference (DID) method to test the impact of an online teaching intervention on students’ LPCFL and its path of action. It aimed to find an effective method for optimizing college students’ foreign language learning effects while considering the endogenous problems among several influencing factors.

Three main research contributions were made in this study. Firstly, the study organically links macro support policies and micro-learning behaviors in the COVID-19 context and proposes an exogenous event as an online teaching intervention to comprehensively investigate the relationship between macro intervention policies and learning behaviors. This is not only helpful in clarifying the effects of macro policies on students’ learning behaviors and the related action mechanism but also provides evidence regarding the micro-learning performance consequences of the examined online teaching intervention. Secondly, this study contributes to the research on factors that influence students’ foreign language learning. LPCFL is influenced by several factors, including teaching interventions, internal class governance, and external policy support. This study examined the impact of an online teaching intervention on students’ LPCFL, indicating that macro policy is also an important factor affecting learning effectiveness. Thirdly, at the micro level, this study can provide a novel research perspective for macro policy implementation entities. The findings indicate that the online teaching intervention expands learning support through corresponding policy intervention, while improving learning input in the implementation process (Cho et al., 2017). Further, the transmission mechanism of the online teaching intervention is fully revealed: introduction of the online teaching intervention → increased policy support → increased student input (support) → improved LPCFL. Additionally, from an online teaching perspective, this study also explains the discipline-level differences in students’ LPCFL and the polarization effect on the policy organization path. This further enhances the current understanding of how an online teaching intervention can affect LPCFL and can provide a reference for universities and relevant departments to effectively implement online teaching interventions.

The remainder of this paper is structured as follows. Section 2 provides the literature review and research hypotheses. Section 3 describes the research design. Section 4 presents an empirical examination of the impact of an online teaching intervention on students’ LPCFL from the perspective of students’ learning support and input and the operational mechanism. Section 5 describes the further examination of cross-sectional differences in the impact of the online teaching intervention on students’ LPCFL from the perspectives of discipline and policy organization. Section 6 provides research conclusions and policy recommendations.

## 2. Literature review

### 2.1. Online teaching interventions

Continuous scientific and technological development has led to the new teaching mode of online teaching, also known as online education or online learning. Especially since the COVID-19 outbreak, online teaching has been considered a new method for imparting knowledge and teaching courses through the Internet (Aretio, 2020; Huang and Wang, 2022). Several scholars have noted that online teaching is a process by which all teaching parties can obtain high-quality online learning resources and effectively construct knowledge and skills through interactive network-reliant learning (Adedoyin and Soykan, 2020; Patricia, 2020). Online teaching relies on existing science and network technology to provide a simulated teaching environment, share online resources, and record data timeously to maximize learners’ subjective initiative (Yang et al., 2020). This talent-training activity relies on the Internet and teachers can impart knowledge to learners and improve their skills in a planned and purposeful way (Long and Zhang, 2014). Online teaching move traditional courses to online platforms and perform corresponding adjustments to the online teaching context (Ravenna et al., 2012). However, teachers often lack higher requirements of online education technology capabilities (Bao et al., 2021; Bardach et al., 2021). Although the total amount of students’ free time has increased, the time students spend on entertainment, especially WeChat, QQ, microblogs, and other online social platforms, has created a significant crowding-out effect on academic time input, reducing it by 15.17% (Mokel and Canty, 2020). In addition, online teaching can make it difficult to create a good atmosphere for peer learning (Kuo et al., 2014; Alamettala and Sormunen, 2020); therefore, colleges and universities must organize online teaching interventions (Garrison and Kanuka, 2004; Pascoe et al., 2022).

Institutions have provided various online teaching interventions, which can be summarized into four categories: online interactive interventions, curriculum structure adjustments, online learning support, and online learning environment interventions. Learning is a process of constructing knowledge in social situations (Bao, 2020), rather than passive acceptance. The essence of online teaching is a learning modality that separates teachers and learners in time and space (Simonson et al., 2009). Adequate and effective interactions are key to improving the effectiveness of online teaching (Moore, 1989; Ally, 2004; Offir et al., 2008). Existing studies have noted that interactive online interventions will significantly promote effectiveness in the online teaching process (Swan, 2001) as well as improve teachers’ guidance and feedback to promote benign teacher-student interactions, form a positive learning atmosphere, and set positive self-behavior expectations, thereby improving online teaching effectiveness (Eom et al., 2006; Elnashar, 2018).

Online teaching media is not a factor that directly promotes students’ learning. By contrast, based on the characteristics of Internet digital media, the design and adjustment of a flexible online curriculum structure are crucial for ensuring the effectiveness of online teaching (Clark, 2001). Digital media development enables the provision of visual and dynamic teaching practices in virtual reality—a way of expressing and presenting that traditional paper, books, and other media cannot achieve. Online teaching can design and organize a better learning experience and create a unique learning environment based on specific teaching content and with the help of digital technology (Rapanta et al., 2020). Therefore, organizational interventions focus on promoting the formation of flexible curriculum structures, appropriate teaching methods, visually appealing content layouts, interactive teaching design, and organized and structural hints in the teaching process, to promote student reflections on learning, facilitate in-depth learning, and improve the effectiveness of online teaching (Dabbagh, 2007).

In the online teaching process, organizations need to provide strong learning support that primarily comprises two elements: instructional and technical support. Instructional support refers to teachers providing online learning materials and feedback to help students perceive changes in teaching methods and their environment in teacher-student interactions and adapt as quickly as possible (Kang and Im, 2013; Leung et al., 2021). By contrast, technical support refers to ensuring the smooth implementation of online courses through various technical means, creating a student-centered learning environment (Revere and Kovach, 2011), improving students’ academic participation, increasing teacher-student interactions (Bernard et al., 2009), and strengthening peer collaboration between students to improve online teaching effectiveness (Borokhovski et al., 2012). The effectiveness of online teaching not only depends on students’ individual characteristics but is also significantly affected by the online learning environment (Thurmond et al., 2002). Therefore, the online learning environment in which an intervention is implemented is another powerful condition, and factors such as network fluency, platform stability, and strong information technology infrastructure are prerequisites for online learning (Ayeb-Arthur, 2017; Rapanta et al., 2020). Thus, the following hypothesis is proposed:

*H1*: Online teaching interventions have a positive effect on LPCFL.

### 2.2. Formative mechanism of the impact of online teaching interventions on LPCFL

Educational researchers have paid close attention to the topic of teaching effects. Through comparative research, several scholars have argued that online and traditional classroom teaching overlap in teaching principles and have no significant differences in learning effects (Clark, 1994; Carter, 1996; Means et al., 2013). Online teaching should be carefully designed to integrate learning objectives, specific learning activities, and measurable results (Muilenburg and Berge, 2001; Oblinger and Hawkins, 2006). Since the 1980s, quality evaluations of teaching and students’ learning effects have gradually become an important trend in research on colleges and universities (Li, 2021). Among various factors that affect LPCFL, most scholars argue that the characters of college students are more decisive (Rapanta et al., 2020; Shao et al., 2020). Among the characteristics that influence individual differences among college students, controllable factors such as learning motivation, ideas, and strategies, are key compared with uncontrollable factors such as intelligence, personality, and learning ability (Wu and Li, 2020). Among various factors, students’ learning input and support are crucial in determining the quality of online learning (Denovan et al., 2019; Rao and Wan, 2020).

Mosher and Mac Gowan (1985) were the first to propose the theory of learning input, positing that learning input, referring to an individual’s full energy, flexibility, and positive emotions, was the embodiment of learners’ understanding of the learning essence and immersion in the learning process. Learning input refers to the time and energy students spend on educational activities (Kuh et al., 2007; Guo and Gao, 2022). Students’ learning input indicates their degree of engagement, in which students actively participate in the learning process (Reeve, 2012) and experience positive emotions (Bao and Zhang, 2012; Osher et al., 2016); moreover, it reflects the combined interaction and influence of dimensions such as behavior, emotion, and cognition (Pauline et al., 2012). Learning input is closely related to learning effects. If students can fully participate in learning, their academic performance will improve (Broadbent and Poon, 2015). However, online and traditional learning have substantial differences. In addition to the skills required to cope with traditional learning, students must quickly adapt to online teaching scenarios, use network resources to achieve autonomous learning, and cultivate ways of thinking and problem-solving abilities under information conditions (Johnson and Sinatra, 2013).

Learning input in foreign language learning is a multidimensional construct, specifically referring to the degree of effort or investment students make in language knowledge and skills and related knowledge in the learning process (Guo and Liu, 2016; Yang et al., 2020). The most important variables positively and directly affecting foreign language learning effectiveness include behavioral, cognitive, emotional, and social input (Liu and Guo, 2021; Zhang, 2022). However, at present, overall learner participation (Yang et al., 2020) and course completion rates (Rao and Wan, 2020) are low for online learning, which will inevitably impact the effectiveness of online teaching and learning. Students most directly experience online teaching; thus, their learning input is the basic element for promoting their active learning and improving online teaching effectiveness (Webber et al., 2013; Kahu and Nelson, 2018). Overall student learning input can be measured by physical and psychological inputs (Astin, 1970, 1997; Zimmerman and Schunk, 2011), which are important factors in predicting teaching effectiveness and academic achievement. Compared with traditional offline teaching, in online teaching, students must have higher self-directed and self-learning abilities; they are constrained by space barriers as students cannot meet in groups and often learn from their bedrooms (Otter et al., 2013). The students’ peer interaction frequency is far lower than the interaction between teachers and students, as well as that between individual students and teaching content (Kuo et al., 2013; Wang, 2019). In online teaching situations, college students’ academic input at the behavioral and cognitive levels has been significantly improved (E and Shen, 2020); however, shortcomings have been noted, such as poor teacher-student communication, weak independent learning abilities among students, and insufficient student participation (Zhang and Hao, 2019; E and Li, 2020), making students more likely to experience the virtual absence state of “online but not studying.” The lack of high-quality independent input from students largely restricts the effectiveness of online teaching (Chen and Cao, 2020).

Learning support is an important factor in improving LPCFL (Dai, 2021; Li et al., 2021) and generally includes two levels. Firstly, it can be provided externally, such as through college organizations and concern from teachers. This can aid students’ academic performance, spiritual affirmation, and encouragement, which are the main social supports that students receive in schools (Ghaith, 2002; Dewaele and Alfawzan, 2018; Jiang and Dewaele, 2019). Specifically, it can be divided into the two dimensions of emotional and professional support. Factors such as effectively providing support, improving college students’ participation in foreign language learning, increasing the depth of students’ learning experiences, and stimulating students’ learning motivation are key to improving LPCFL through external support (Li et al., 2019; Liu et al., 2021). In general, the more learning support students perceive, the higher their online learning performance will be (Li et al., 2018; Rao and Wan, 2020). Therefore, increasing support for students’ learning through online teaching is crucial to improve online students’ participation and learning effectiveness (Lei et al., 2018). Secondly, students can provide internal support *via* emotional and behavioral support for their own online learning, which is also the most direct expression of their learning willingness and interest (Li, 2020; Tan and Fu, 2020). When students perceive external support from their colleges and teachers, they will also show higher levels of internal learning interest and support (Zhang, 2012; Wang and Jiang, 2022), more actively participate in teaching activities, and increase their learning input (Fisher et al., 2021). Additionally, external learning support can improve students’ learning adaptability, which is positively related to their learning interests, classroom participation, and academic performance (Ghaith et al., 2007; Piniel and Albert, 2018; Liu and Guo, 2021). Thus, based on the above, the following hypothesis is proposed:

*H1*: Learning input and learning support are the intermediary paths between online teaching interventions and LPCFL.

## 3. Materials and methods

### 3.1. Sampling

This study aims to understand the impact of an online teaching intervention on LPCFL. Quarantine procedures vary across different countries, and the COVID-19 pandemic has influenced people’s psychological characteristics. The differences in cultures and epidemic prevention methods in different countries are subject to different psychological interventions. As it would be impracticable to include a sample from each country, we collected samples from one college in Mainland China. We adopted a purposive sampling method and established several conditions during sampling to ensure the representativeness of the research sample. First, we selected Mainland China as the main area for sampling, as this was where the pandemic was initially the most severe, and it had the strictest quarantine policies. Thus, the sample is representative to a certain extent. Second, we focused on teachers and students to understand the status of online teaching intervention; thus, colleges were selected as the main sampling context. As a typical strict control intervention mode, China has chosen Chinese Mainland as the main sampling area, because it is the most severely affected area and has the strictest quarantine policy. All universities in China have adopted the model of reducing offline teaching and increasing online teaching. Therefore, using a single university as a sample is also representative. Students’ evaluations may be incomparable because of the differences in the degree of difficulty and boredom of the courses in varied disciplines at different universities. Therefore, English teaching implemented in the whole school is selected as the research sample, excluding all other courses in the school, including the behavior of the administrative department to accurately collect representative samples.

Further, we collected data from the same respondents each semester from teacher evaluation records, student learning records, and student questionnaires, which may have led to common method bias. Accordingly, we used Harman’s single-factor verification test and the DID method to analyze all the measurement items using a non-rotating matrix.

### 3.2. Scale development

The back translation method was applied to construct the measurement for this study. First, the researchers invited an expert proficient in both Chinese and English to translate the original English scale items into Chinese and then invited another bilingual expert to translate the Chinese version into English without knowing the original scale. A scholar in the education field conducted the final review to ensure that the meaning expressed by the questions remained consistent with the original scale (i.e., between the original English questionnaire and the translated English questionnaire).

In view of the structural changes in students’ learning input between online and offline teaching situations, previous research has typically adopted the two categories of students’ skill and manifestation input. The former includes five indicators: completing assignments on time (S11), time invested in learning (S12), listening conscientiously (S13), obeying classroom discipline (S14), and consulting teachers individually after class (S15). The differences in students’ learning time investment have been found to lead to significant differences in their perceptions of online and offline teaching effectiveness (Hiltz et al., 2000). Therefore, we included students’ time investment and other inputs to explore the changes in students’ skill input across different teaching situations. In selecting the two intermediary dimensions of students’ learning input and learning support, this study was based on the online learning input and support scale compiled by Marcia and Dixson (2010). The dimensions of students’ learning input and support were determined referring to the relevant indicators in the learning input and support questionnaire designed by Gunuc and Kuzu (2015) and the learning input and support evaluation scale designed by Hamish and others (Coates, 2007). The former measure includes skill input and learning expression input, whereas the latter includes emotional and interactive support.

The “learning input” includes “skills input” and “manifestation input”; the former has five parts, which are “complete assignments on time,” “time invested in learning,” “listen conscientiously,” “obey classroom rules,” and “consult teachers individually after class.” The latter includes three parts, which are “online and offline learning are considered to be the same,” “actively seek out learning resources for self-directed learning,” and “overall satisfaction with the e-learning results.” “Learning support” includes “emotional support” and “interactive support”; the former includes four parts which are “think online learning is quite helpful,” “be willing to study with classmates,” “be willing to share opinions in online courses,” and “not afraid of learning difficulties” The latter includes five parts, which are “participate in class discussions,” “gains in the discussion,” “sense of belongingness,” “will pay attention to others,” and “discuss course-related issues with classmates after class.” Each part is scored freely with a full score of five points. Finally, several parts are added to get the total score. For comparability, the total score is changed into a percentage system.

### 3.3. Survey design and administration

We referred to self-reported measures used in previous studies to assess students’ LPCFL (Al-Azmi, 2018). The measure for the online learning invention was compiled with reference to Ajzen (1991) and Khzam and Lemoine (2021) and comprised two measurement items. The measurement of learning input and learning support were compiled referencing Ajzen (1991) and included two measurement items. The measurement of learning input was compiled referencing Bagozzi and Pieters (1998) and used to evaluate the emotional responses of students to achieve performance goals set. Learning support was measured using items developed by Bagozzi and Lee (2002), which evaluated the useful environment and students’ sense of identity with online teaching.

Additionally, existing research has shown that the teaching effectiveness of online courses is heterogeneous. A comparison of online and traditional offline learning showed that gender is an effective moderator of online teaching effectiveness (Figlio et al., 2013). Another study found that in a traditional offline model, the performance of female students was significantly lower than that of male students. However, with online learning, no significant gender differences were observed (Brown and Liedholm, 2002). In addition, some differences have been reported in evaluations of online course teaching effectiveness by students with different majors and in varied school years (Chen and Jia, 2020). Similar majors in Chinese universities are managed uniformly in one department; therefore, we include relevant variables in this study, such as students’ individual characteristics, department differences, and school years as controls to improve the reliability of the findings. Table 1 summarizes the relevant variables used and their operational definitions including the sub-items for each variable.

**Table 1:** Relevant variables and their operational definitions.

Variables	Variable name	Index	Operationalization
Dependent variable	Learning performance	Learning attainment	Satisfaction score (students evaluate according to 5 points into full scores)
Independent variable	Teaching intervention	Whether to intervene	Intervention = 1, No intervention = 0
	Time	Before and after the COVID-19 outbreak	Before COVID-19 = 0, After COVID-19 = 1
Intermediary variables	Study input (studyin)	Skills input (S1)	Complete assignments on time(S11), time invested in learning (S12), listen conscientiously (S13), obey classroom rules (S14), consult teachers individually after class (S15)
		Manifestation input (S2)	Online and offline learning are considered to be the same (S21), actively seek out learning resources for self-directed learning (S22), overall satisfaction with the e-Learning results (S23)
	Study support (studysu)	Emotional support (S3)	think online learning is quite helpful (S31), be willing to study with classmates (S32), be willing to share opinions in online courses (S33), not afraid of learning difficulties (S34)
		Interactive support (S4)	Participate in class discussions (S41), gains in the discussion (S42), sense of belongingness (S43), will pay attention to others (S44), discuss course-related issues with classmates after class (S45)
Control variables	Individual characteristics	Gender	Dummy variable, male = 1, female = 0
		Year	Freshman = 1, Sophomore = 2, Junior = 3, Senior = 4
		School	Dummy variables, 15 schools, evaluate 1 to 15 respectively

### 3.4. Demographic profile

This questionnaire was collected by the college’s Office of Academic Affairs through compulsory means. As it involves students’ achievement, it was collected comprehensively, and the 142,151 survey results obtained were valid to our study. The statistical characteristics of the survey results are shown in Table 2.

**Table 2:** Demographic characteristics of respondents (*n* = 142,151).

Variable name	Index	Operationalization	Number of samples	percentage
Learning performance	Learning attainment	/	/	/
Teaching intervention	Whether to intervene	Intervention	78,325	55.1%
		No intervention	63,825	44.9%
Time	Before and after the COVID-19 outbreak	Before COVID-19	60,272	42.4%
		After COVID-19	81,879	57.6%
Individual characteristics	Gender	Male	61,267	43.1%
		Female	80,884	56.9%
	Year	Freshman	77,330	54.4%
		Sophomore	55,438	39.0%
		Junior	8,529	6.0%
		Senior	852	0.6%
	School	School of Business Administration	10,093	7.1%
		School of Finance, Taxation, and Public Administration	11,372	8.0%
		School of Accounting	18,764	13.2%
		School of International Business and Economics	8,245	5.8%
		School of Economics	6,397	4.5%
		School of Finance	12,367	8.7%
		School of Statistics	8,245	5.8%
		School of Information Management	8,956	6.3%
		School of Tourism and Urban Management	4,975	3.5%
		School of Law	5,970	4.2%
		School of Software and Internet of Things Engineering	12,651	8.9%
		School of Foreign Languages	5,117	3.6%
		School of Humanities	4,975	3.5%
		School of Art	8,386	5.9%
		School of Physical Education	1,705	1.2%
		School of International	13,930	9.8%
		School of Virtual Reality (VR) Modern Industry	3	0.0%

As shown in Table 2, these answers show good diversity and stability. The distribution of participants’ gender and grade is relatively stable. The number of participants before and after the epidemic and the number of students receiving intervention and not receiving intervention is the same.

### 3.5. Methodology

We used the difference in difference (DID) approach to test this study’s research hypotheses. The substitution variable approach was applied using State 16.0 to confirm reliability and validity. In recent years, DID models are mostly used for quantitative evaluation of the implementation effect of public policies or projects in each field. Compared with other methods, the DID model is suitable for this study because the research objective is exploratory research for theory development, the analysis is for assessment purposes, the model is complex and includes one or more formative constructs. Thus, DID can determine the effect of the online intervention implementation.

Regarding the benchmark model, the following model was designed according to the principle of the DID model to identify the effects of an online teaching intervention on LPCFL:


(1) $${\mathrm{perform}}_{\mathrm{it}}=\theta \left({\mathrm{treat}}_{\mathrm{it}}*{\mathrm{post}}_{\mathrm{it}}\right)+\beta x_{\mathrm{it}}+\lambda_{t}+\mu_{i}+\varepsilon_{\mathrm{it}}$$

where i, t, and j represent students, semesters, and colleges, respectively. Performance refers to students’ LPCFL, which was measured using self-assessment. Treat is the intervention grouping variable; when treat equals 1, it represents the intervention group, and when treat equals 0, it represents the non-intervention group. Post is a time grouping variable because Chinese universities have two semesters: spring (March–mid-July) and autumn (September to mid-January of the next year). The value of 3 semesters from the autumn semester of 2018 to the autumn semester of 2019 is 0. The value of 4 semesters from the spring semester of 2020 to the autumn semester of 2021 is 1. X represents the control variables. Referring to existing research, we controlled for students’ department of study, school year, and gender. The 15 departments were represented by dummy variables of 1–15. The school year was expressed as years 1–4. Student gender (gender) was represented by a dummy variable (male = 1, female = 0). Time-fixed effects described the samples in greater detail than time grouping (post) and individual-fixed effects described the samples in greater detail than student grouping (treat). Therefore, adding treat and post items to model (1) was unnecessary. Instead, we only added their cross items; thus, model (1) is actually a DID model based on two-way fixed effects. λ_t_ represents time-fixed effects, μ_i_ represents students’ individual fixed effects that did not change with time, and *ε* represents a random disturbance term. According to the principles of the DID model, this study focused on the cross-term treatment*post coefficient of post θ, which represents the influence of the online teaching intervention on students’ LPCFL after excluding other potential interference factors. In addition, because of the adaptability of teachers and students to the intervention policy during the COVID-19 pandemic and the lag and timeliness of the intervention effect, we had reason to believe that the impact of the online teaching intervention on students’ LPCFL would be non-linear (Latif, 2022).

Next, we examined the path from the online teaching intervention to its effects on students’ LPCFL. This study proposes that, because of differences in the learning basis and requirements of students with different majors (although these students have different choices in learning models), the path of foreign language learning achievements will ultimately be reflected in learning input and learning support. Thus, this study examined whether the online teaching intervention affected students’ LPCFL with respect to learning input and learning support. To design a model to test this empirically, we referred to Li and Zheng (2016), and Fan and Peng (2004), and included regulatory variables in the benchmark model to test for the significance of the impact mechanism. The specific model design is as follows:


(2) $$\begin{array}{c} {\mathrm{studyin}}_{\mathrm{it}}=\theta \left({\mathrm{treat}}_{\mathrm{it}}*{\mathrm{post}}_{\mathrm{it}}\right)+\beta x_{\mathrm{it}}+\lambda_{t}+\mu_{i}+\varepsilon_{\mathrm{it}}\\ {\mathrm{studysu}}_{\mathrm{it}}=\theta \left({\mathrm{treat}}_{\mathrm{it}}*{\mathrm{post}}_{\mathrm{it}}\right)+\beta x_{\mathrm{it}}+\lambda_{t}+\mu_{i}+\varepsilon_{\mathrm{it}} \end{array}$$

(3) $$\begin{array}{c} {\mathrm{perform}}_{\mathrm{it}}=\theta {\mathrm{studyin}}_{\mathrm{it}}+\beta x_{\mathrm{it}}+\lambda_{t}+\mu_{i}+\varepsilon_{\mathrm{it}}\\ {\mathrm{perform}}_{\mathrm{it}}=\theta {\mathrm{studysu}}_{\mathrm{it}}+\beta x_{\mathrm{it}}+\lambda_{t}+\mu_{i}+\varepsilon_{\mathrm{it}} \end{array}$$

where perform refers to LPCFL and learning input and learning support are intermediary variables. Existing research has mostly examined students’ learning input and support levels using questionnaires and their usual performance (Zhang et al., 2014; Guo et al., 2018). Compared with the questionnaire, usual performance is timelier and more realistic, and can better reflect a student’s commitment behavior and willingness. Therefore, we used a combination of questionnaire responses and usual performance as proxy variables for learning input and learning support. The other variable definitions are the same as in Model (1).

### 3.6. Data analysis

This study uses questionnaire surveys to determine the research sample; therefore, it is necessary to test the reliability and validity. At the same time, to ensure the accuracy of the test, this study further uses Stata 16.0 for confirmatory factor analysis (CFA). Data test results show that Cronbach’s α of all constructs in Table 3 are higher than 0.8. According to the research standard, when the coefficient of Cronbach’s α exceeds 0.7 (Cao et al., 2022), the reliability is significant. AVE and CR of all dimensions in Table 3 are higher than the recommended values of 0.5 and 0.8; thus, all dimensions in this study have good convergence effectiveness.

**Table 3:** Scale measurement.

	1	2	3	4	5	6	7	8
1. Learning performance	0.712							
2. Skills input (S1)	0.298^**^	0.815						
3. Manifestation input (S2)	0.842	0.250^**^	0.766					
4. Emotional support (S3)	0.796	0.306^*^	0.592^*^	0.877				
5. Interactive support (S4)	0.443^*^	0.625	0.672	0.424^*^	0.914			
6. Gender	0.924	0.126^**^	0.606	0.849	0.298^**^	0.859		
7. Year	0.829	0.460	0.250^**^	0.798	0.422^**^	0.756	0.891	
8. School	0.255^**^	0.144^**^	0.765	0.223^**^	0.386^**^	0.339^**^	0.337^**^	0.923
Cronbach’s α	0.831	0.829	0.812	0.898	0.835	0.813	0.824	0.875
AVE	0.896	0.845	0.891	0.872	0.818	0.816	0.792	0.769
CR	0.882	0.914	0.869	0.917	0.874	0.902	0.866	0.897

** *p* < 0.01.

* *p* < 0.05.

Using the collected data, we conduct a univariate t-test analysis on all samples to preliminarily explore the differences in students’ LPCFL before and after the intervention implemented in response to the COVID-19 outbreak. The purpose is to determine whether the students’ LPCFL in the treatment group was influenced by the intervention as posited by this study. In addition, the impact and its mechanism are also assessed using DID analysis. Thereafter, heterogeneity regression analysis is conducted to examine the effects of the students’ individual character dimensions on teaching performance.

According to the classification method described above, all samples were divided into the intervention (treatment) and non-intervention (control) groups. The treatment and control groups reflected the LPCFL of students who had and had not received the online learning intervention, respectively. Prior to COVID-19, there were three semesters from the autumn semester of 2018 to the autumn semester of 2019, and after the COVID-19 outbreak, there were four semesters from the spring semester of 2020 to the autumn semester of 2021. The results are shown in Table 4.

**Table 4:** Online learning intervention and students’ foreign language learning performance: Univariate *t*-test results.

	Treatment group (1)	Control group (2)	Difference (1)–(2)	*t*-test (1)–(2)
Before COVID-19	22.75452	22.65581	0.0987	13.0242^***^
After COVID-19	22.97826	22.71864	0.2596	10.2668^***^

*** *p* < 0.01.

As Table 4 shows, prior to COVID-19, a minor difference in LPCFL existed between the treatment and control groups. Specifically, the average LPCFL of students in the treatment group was 0.0987 scores higher than that of the control group (the significance level was 1%, with a corresponding *t*-value of 13.0242). This indicated that prior to the COVID-19 outbreak, the LPCFL of the students in the treatment group was not significantly higher than that of the control group. This was because the school used for the study site had a course selection system that allowed students to choose their teachers. Further, the students in the treatment group were relatively active learners. However, although differences existed among students in course selection classes, the difference was minor. After the COVID-19 outbreak, generally, students in the treatment group showed high learning performance with strong comprehensive advantages and tended to “choose the courses by good teachers” and receive intervention and support from the school (Jiang et al., 2021). After the implementation of the online learning intervention, the average LPCFL of the students in the treatment group remained significantly higher than that of those in the control group (the significance level was 1%, with a corresponding *t*-value of 10.2668), and the difference between the two increased from 0.0987 before the online learning intervention to 0.2596 post the intervention. This shows that the online learning intervention indeed played a significantly positive role in improving the LPCFL of the students in the treatment group, widening the gap between the treatment and control groups. However, whether this gap is statistically significant required further testing using the DID model.

## 4. Results

### 4.1. Parallel trend test

According to the univariate analysis of students’ LPCFL, implementing the online learning intervention widened the LPCFL gap between students in the treatment group and those in the control group by more vigorously boosting the LPCFL of students in the treatment group. However, this does not substantively prove that the online learning intervention can promote students’ LPCFL for two reasons. First, whether this gap is statistically significant is unknown. Second, univariate analysis can neither control for other individual variables that affect students’ LPCFL nor exclude interference from other influencing factors on the estimation results. Therefore, to estimate the impact of the online learning intervention on students’ LPCFL more accurately, we further controlled for students’ characteristic variables, individual fixed effects, and time-fixed effects and adopted a more rigorous DID model for estimation analysis. The results are shown in Table 5.

**Table 5:** Online teaching intervention and students’ LPCFL: DID results.

	(1)	(2)	(3)
Post	0.2046^***^(0.0099)		0.0646^**^(0.0319)
Treat	0.1479^***^(0.0165)		0.0889^***^(0.0208)
Treat*post		0.2335^***^(0.0097)	0.1550^***^(0.0335)
Gender	0.0001(0.0007)	0.0000(0.0007)	0.0001(0.0007)
Year	0.0956^***^(0.0076)	0.0964^***^(0.0075)	0.0952^***^(0.0076)
Department	0.0005(0.0009)	0.0005(0.0009)	0.0003(0.0009)
C	22.5608^***^(0.0176)	22.6849^***^(0.0121)	22.6139^***^(0.0210)
R^2^	0.0054	0.0054	0.0056
Obs	142,151	142,151	142,151

***p < 0.01, **p < 0.05.

The regression results in Table 5 show that, after controlling for students’ characteristic variables, the coefficients of the treatment items, post items, and treatment*post items of concern in this study were positively significant at the 1% level. This indicates that, compared with the students in the control group, the online learning intervention significantly improved the LPCFL of the students in the treatment group. Thus, the online learning intervention substantially boosted students’ LPCFL, and H1 cannot be statistically rejected.

### 4.2. Main intervention effects on LPCFL

Based on a quasi-natural experiment of an online teaching intervention, this study used the DID model to investigate the impact of the intervention on students’ LPCFL.

#### 4.2.1. Placebo test

**Figure 1:**
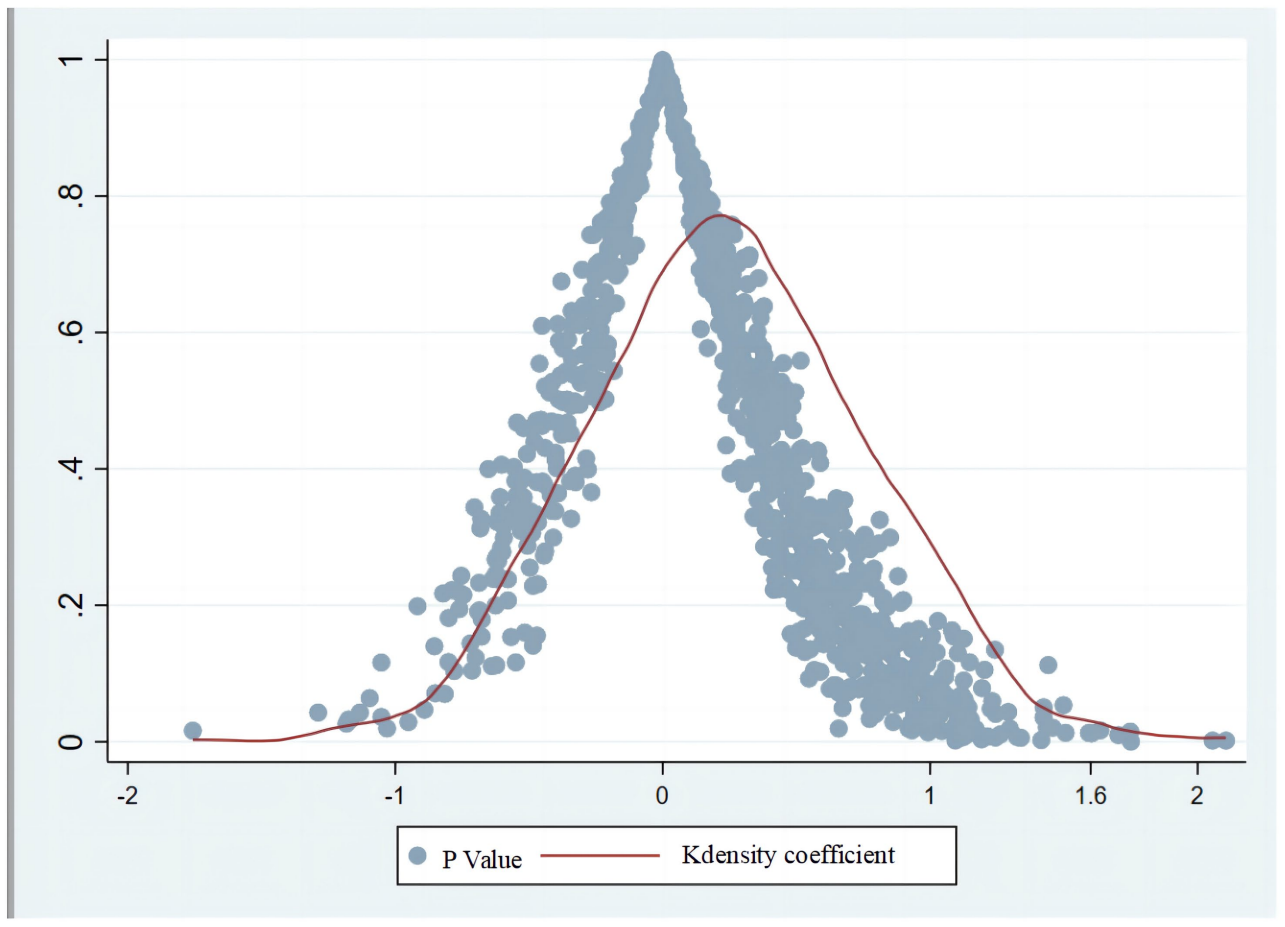
Random impact coefficient Kdensity and corresponding *p*-value.

To ensure the robustness of the research findings, we conducted a placebo test by constructing a false treatment group and false intervention year to further verify the impact of the online teaching intervention on students’ LPCFL and confirm whether it was affected by missing variables. Referring to Liu et al. (2022), we tested the correctness of the intervention object of online teaching using DID for a constructed false handling group. Among the samples in this study, the students had learned foreign languages for seven semesters. Each semester included approximately 20,000 samples; 10,000 samples were randomly selected and a kernel density map of the regression coefficients of 1,000 random shocks and the corresponding *p*-value distribution were created (Figure 1).

Figure 1 shows that the estimated values of the randomly generated variable samples impacted by COVID-19 are generally concentrated around 0, the *p*-values of the estimated values are large, and most of the variables are not significant; thus, the test did not passed. This indicates that the study’s results are not affected by missing variables. As suggested by Armstrong and Overton (1977), the verification check, DID, was conducted to determine whether significant differences were present between the two groups. The verification results showed a significant difference in the LPCFL between the two groups, indicating that the problem of non-response bias was not significant. The analysis results demonstrate that the implementation of the online teaching intervention during the COVID-19 pandemic significantly improved the LPCFL of the participants, and this conclusion remained valid after a placebo test was used to control for potential problems with endogeneity. Therefore, no common method bias was present in this study.

#### 4.2.2. Robustness test

To further verify the stability of this conclusion, the proxy variable was replaced again. Specifically, students’ actual final exam scores were used instead of LPCFL. Table 6 shows the results of the robustness test through substitution variables.

**Table 6:** Robustness test of substitution variables.

	(1)	(2)	(3)
Post	0.2039^***^(0.0099)		0.0635^**^(0.0318)
Treat	0.1487^***^(0.0163)		0.0889^***^(0.0208)
Treat*post		0.2331^***^(0.0097)	0.1554^***^(0.0335)
Gender	0.0001(0.0006)	0.0002(0.0019)	0.0001(0.0012)
Year	0.0964^***^(0.0076)	0.0973^***^(0.0075)	0.0960^***^(0.0076)
Department	0.0002^***^(0.0001)	0.0003^***^(0.0001)	0.0003^***^(0.0001)
C	22.5445^***^(0.0174)	22.6694^***^(0.0107)	22.5956^***^(0.0206)
R^2^	0.0055	0.0055	0.0056
Obs	142,151	142,151	142,151

***p < 0.01, **p < 0.05.

The regression results in Table 6 show that the online learning intervention significantly improved the LPCFL of the students in the treatment group after the alternative index of LPCFL was applied. This indicates that the conclusion is robust and H1 cannot be statistically rejected.

### 4.3. Moderating effects of learning input and learning support

Regarding the path through which the online teaching intervention can boost students’ LPCFL, the theoretical analysis indicates that, although different students have different foreign language learning endowments and technologies, the path to improving student performance will ultimately be reflected in learning input and support. Thus, whether learning input and support are factors through which the online teaching intervention can boost students’ LPCFL needs to be determined. Therefore, we tested the mediating effects of learning input and support in the relationship between the online teaching intervention and students’ LPCFL. Learning input and support were each expressed from two aspects: learning input includes skill input (S1) and expression input (S2), and learning support includes emotional support (S3) and interactive support (S4). The maximum variance inflation factor value in the models was 1.665, which was less than 3.3, indicating that collinearity was not a problem in this study (Petter et al., 2007; Table 7).

**Table 7:** Mediation effect test.

Stage I: Independent variable-intermediate variable				
	S1	S2	S3	S4
Treat*post	0.1212^***^(0.0052)	0.1186^***^(0.0049)	0.1239^***^(0.0063)	0.2459^***^(0.0095)
Gender	0.0000(0.0009)	0.0001(0.0009)	0.0003(0.0017)	0.0002(0.0019)
Year	0.0352^***^(0.0040)	0.0282^***^(0.0038)	0.0127^***^(0.0049)	0.0948^***^(0.0074)
Department	0.0001^**^(0.0000)	0.0001^**^(0.0000)	0.0001(0.0001)	0.0002^**^(0.0001)
C	13.7117^***^(0.0057)	13.7475^***^(0.0054)	18.4277^***^(0.0069)	22.6769^***^(0.0104)
R^2^	0.0046	0.0047	0.0029	0.0062
Obs	142,151	142,151	142,151	142,151
Stage II: Intermediate variable-dependent variable				
	(5)	(6)	(7)	(8)
S1	1.6693^***^(0.0023)			
S2		1.6909^***^(0.0028)		
S3			1.2455^***^(0.0024)	
S4				0.9516^***^(0.0010)
Gender	0.0001(0.0009)	0.0002(0.0013)	0.0005(0.0019)	0.0005(0.0031)
Year	0.0394^***^(0.0035)	0.0505^***^(0.0040)	0.0841^***^(0.0045)	0.0068**(0.0028)
Department	0.0008**(0.0000)	0.0017***(0.0004)	0.0027(0.0005)	−0.0002(0.0003)
C	−0.2052***(0.0322)	−0.5660***(0.0387)	−0.2536***(0.0458)	1.0965***(0.0232)
R^2^	0.7865	0.7246	0.6461	0.8645
Obs	142,151	142,151	142,151	142,151

***p < 0.01, **p < 0.05, *p < 0.1.

The results of process model 7 show that the mediating effect of learning input and support was significant, indicating that the online teaching intervention promoted learning input and learning support. Compared with learning input, the online teaching intervention better promoted learning support; however, both learning input and support could effectively promote students’ LPCFL. Although learning support may have less of a promotion effect on students’ LPCFL compared with learning input, both play a strong intermediary role, which supports H2.

## 5. Influence of the online teaching intervention on LPCFL: A heterogeneity test

The above empirical results show that the online teaching intervention explored in this study significantly improved students’ LPCFL. To test whether students in different majors and organization types can improve their LPCFL through an online teaching intervention, we further divided the students of the treatment group into three groups—humanities, science and engineering, and economics and management disciplines according to students’ majors—and simultaneously, divided the students of the treatment group into four groups by the organization type: “college teaching management: teachers,” “college teaching management: students,” “student management groups: teachers,” and “student management groups: students.” Here, the student grouping variable “treat” differed from the setting in model (1), in that “treat = 0″ still represented the control group in model (1), but “treat = 1″ no longer represented all students affected by the online teaching intervention. Instead, it represented students of different majors and intervention organization types.

### 5.1. Test based on discipline characteristics

Through a text analysis of the students’ major subjects, this study divided the treatment group in the original model (1) into three groups (i.e., humanities group, science and engineering group, and economics and management group) according to the students’ majors. Among the 15 colleges, the humanities group included the School of Physical Education, School of Humanities, School of Foreign Languages, School of Law, and School of the Arts; the science and engineering group included the School of VR Modern Industry, School of Statistics, School of Software and Internet, and School of Information Management; and the economics and management group included the School of Accounting, School of Finance, School of Finance and Taxation, School of Business Administration, School of International Business and Economics, School of Economics, and School of Tourism Management. The regression results are shown in Table 8.

**Table 8:** Discipline heterogeneity effect test.

	(1)	(2)	(3)
Treat*post	0.3216^***^(0.0252)	0.1123^***^(0.0237)	0.2555^***^(0.0118)
Gender	0.0001(0.0011)	0.0003(0.0009)	0.0001(0.0012)
Year	0.1012^***^(0.0206)	−0.0115(0.0164)	0.1441^***^(0.0094)
Department	−0.0440^***^(0.0082)	−0.0081^***^(0.0024)	0.0021^*^(0.0011)
C	23.3678^***^(0.1327)	22.8046^***^(0.0307)	22.6581^***^(0.0137)
R^2^	0.0099	0.0013	0.0079
Obs	20,948	26,247	94,956

***p < 0.01, *p < 0.1.

For the estimated results of the heterogeneity effect test in Table 8, Columns (1)–(3), list the estimated results on whether the online teaching intervention was able to boost the LPCFL of students in the humanities, science and engineering, and economics and management groups, respectively. The results indicate that the moderating effect of the online teaching intervention on the LPCFL of students in each discipline group was significantly positive at the 1% level. This supports the theoretical assertion of this study that, through the online teaching intervention, although students’ LPCFL will vary depending on their disciplines, it can ultimately effectively boost students’ LPCFL regardless of their majors. Therefore, H1 is further verified and cannot be statistically rejected.

Notably, from the coefficient value of the intervention effect, the effect values of the online teaching intervention on the LPCFL were 0.3216, 0.1123, and 0.2555 for students in the humanities, science and engineering, and economics and management groups, respectively. It can be predicted that the impact of the online teaching intervention from most to least on LPCFL for students in each discipline group is in the order of the humanities, economics and management, and science and engineering groups. Regarding the reason for this, among the five schools of the humanities group, except for the school of foreign language, students from the School of the Art and School of Physical Education have lower starting points and higher learning requirements for foreign languages. Students from the School of Humanities and School of Law have relatively lower foreign language requirements. Thus, when participating in the online teaching intervention, the latter students had fewer extracurricular activities and more time and attention to devote to learning; thus, more learning input and support would be obtained, and LPCFL would improve faster. The students in the science and engineering group always have higher enrollment and had few extracurricular activities; thus, the potential for learning input and learning support was small Therefore, the online teaching intervention led to the lowest level of improvement in LPCFL for this group.

### 5.2. Test based on intervention organization mode

Through text analysis of the intervention organization mode, we divided the treatment group in the original model (1) into four types: the “teaching management: teachers” group, “teaching management: students” group, “student management: teachers” group, and “student management: students” group. The regression results of the four intervention organization mode heterogeneity effects are shown in Table 9.

**Table 9:** Intervention organization mode heterogeneity effect test.

	(1)	(2)	(3)	(4)
Treat*post	0.1136^***^(0.0170)	0.0883^***^(0.0129)	−0.3487^***^(0.0594)	−3.2778^***^(0.2251)
Gender	0.0001(0.0015)	0.0003(0.0009)	0.0003(0.0022)	0.00052(0.0019)
Year	0.0655^***^(0.0115)	0.0906(0.0100)	0.0471(0.0346)	−0.1798(0.1331)
Department	0.0008(0.0013)	0.0002(0.0012)	−0.0002(0.0039)	−0.0557^***^(0.0187)
C	23.1331^***^(0.0208)	22.7532^***^(0.0167)	22.3274^***^(0.0480)	22.5812^***^(0.2751)
R^2^	0.0031	0.0015	0.0030	0.1820
Obs	20,948	83,212	12,455	1,102

***p < 0.01.

Table 9 shows the estimated results of the intervention organization mode heterogeneity effect, and Columns (1)–(4), respectively, list the estimated results on whether the online teaching intervention could boost students’ LPCFL under different intervention organization modes. The results indicate that the moderating effect of the online teaching intervention on students’ LPCFL was significant at the 1% level and the polarization phenomenon was serious. This supports the theoretical assessment of this study that, through online teaching intervention, although students’ LPCFL differed because of differences in intervention organization methods, the two organization methods from “college teaching management,” which were the “college teaching management-teachers” group and “college teaching management-students” group were significantly positive. Further, the two organization methods from “college student management,” which were the “student management group-teachers” group and “student management group-students” group, were significantly negative.

Next, we determined the coefficient values for the adjustment effect of the online teaching intervention. The effect values of both organization methods for teachers and students downward through college teaching management were 0.1136 and 0.0883, respectively; however, the effect values of both organization methods for teachers and students downward in the student management groups were −0.3487 and −3.2778, respectively. This indicates that the impact of the online teaching intervention was positive through college teaching management but negative through student management groups. This may be because the intervention and communication ability of the teaching management team and the cognition among teachers and students was more authoritative, and the intervention also had a more positive stimulating effect. The student management group focuses more on students’ lives and daily behaviors. During the online teaching intervention, the intervention strength and its ability to be implemented in the student management group would be counterproductive and negatively affected, thereby reducing students’ LPCFL.

## 6. Conclusion

This study used the shift to online teaching during the COVID-19 outbreak in China as a quasi-natural experiment and applied the DID method to investigate the impact of online teaching during the outbreak on students’ LPCFL and the related path of action.

### 6.1. Research conclusion and insights

Focusing on China, this study examined the impact of an online teaching intervention on students’ LPCFL and the related effects of learning input and learning support. We used foreign language learning course selection, classroom learning records, and teaching evaluation results from the last seven semesters, as well as the questionnaire survey of teachers and students during the COVID-19 period and relevant school documents. The findings of this study indicate that the online teaching intervention improved learning input and support which, in turn, increased LPCFL. Therefore, the online teaching intervention influenced students’ LPCFL. The findings indicate that colleges should implement the online teaching intervention and organize it by teacher management terms, which would favorably affect LPCFL in terms of increasing students’ learning input and support. Therefore, this study may serve as a reference to colleges for increasing their students’ LPCFL during the COVID-19 pandemic, based on the conclusions below.

#### 6.1.1. Significant improvements in students’ LPCFL

Using DID regression, after controlling for students’ characteristic variables and the fixed effects of student individuals and students’ time, the analysis showed that, at the statistical level of 1%, the online learning intervention significantly improved the LPCFL of students in the treatment group. Further, after controlling for possible endogeneity through placebo and robustness tests, the conclusions remained valid. The online teaching intervention improved LPCFL by promoting students’ learning input and learning support. Further, the online teaching intervention promoted additional learning support, but learning input better promoted students’ LPCFL. Learning input and support were found to play strong mediating roles, which supports H2.

#### 6.1.2. Disciplinary heterogeneity effect

Under the online teaching intervention, although students’ LPCFL may vary depending on their disciplines—whether they are in the humanities group, the science and engineering group, or the economics and management group can ultimately effectively boost students’ LPCFL. However, from the perspective of the coefficient value of the intervention effect, the effect value of the humanities, science and engineering, and economics and management group students is 0.3216, 0.1123, and 0.2555, respectively. We believe that this conclusion is of great significance because English teaching in China has obvious unity; that is, the same syllabus, textbooks, and teachers, and even sometimes students of different majors will be confused in the same English class, which shows that students of different majors have the same direction of feedback on interference, which is slightly different in intensity. This can be promoted in the future.

#### 6.1.3. Polarization effects of organizational mode

Under different intervention organization modes, the adjustment effects of the online teaching intervention on students’ LPCFL were significant at the 1% level; however, the polarization phenomenon was found to be relatively serious. The two organizational methods of teachers and students downward through the college’s teaching management were positive, with effect values of 0.1136 and 0.0883, respectively. However, the two organizational methods of teachers and students downward through the student management group were negative, with effect values of –0.3487 and –3.2778, respectively.

### 6.2. Theoretical contributions

This study makes novel contributions to intervention theory. First, it validated perceived teaching intervention and organization activities (intervention organization by teaching management terms or student management terms) in the online teaching environment. Second, this study contributes to the current understanding of online teaching interventions and their effects through increasing student input and support on LPCFL in the Chinese context. Teaching theory posits that student performance is derived from students’ own input and support. Similarly, when students gain a new online environment referring to foreign language learning, they access previously learned information and judgment factors to the new class space. Thus, organizing interventions from teacher management terms should improve students’ own input and support and, in turn, increase their performance. Third, this study found that an online teaching intervention could significantly strengthen student performance in China. The study states that throughout the teaching intervention, students invest their learning input and learning support depending on their subjects and organization model, which are then used to appraise achievement. Consequently, online teaching interventions can stimulate learning input and learning support among Chinese students with respect to foreign languages.

Finally, this study showed that the different organization models used in the online teaching intervention resulted in a relatively serious polarization phenomenon. Prior research revealed that interventions can increase students’ willingness to input learning and then enhance performance in China (Bao et al., 2021). Thus, our findings provide novel contributions to the organization model of online teaching.

### 6.3. Managerial recommendations

Based on this study’s findings, we can provide several recommendations for college administrators and teaching managers. First, this study discovered that colleges must have interventions for “online teaching,” which significantly influence students’ learning performance in foreign languages. Therefore, teaching managers should prioritize strengthening their focus on students’ input and support. Second, this study found that students were positively affected by the intervention regardless of their majors. Here, managers should implement interventions according to students’ distinctive disciplinary characteristics. Finally, this study showed that an online teaching intervention could demonstrate a polarization effect between different organization modes. Accordingly, managers may strengthen organization among teachers through teaching management terms to increase students’ willingness to learn, thereby encouraging students to enjoy their foreign language lessons. Thus, online teaching interventions will increase LPCFL and developing such interventions may help students improve their foreign language abilities.

#### 6.3.1. Focus on disciplinary differences and adopt different methods

Students with different majors have distinctive disciplinary characteristics. When implementing an intervention, different approaches should be adopted to improve students’ learning performance according to the characteristics of different disciplines of study.

#### 6.3.2. Strengthen organizational intervention and provide strong support

Under a significant external impact, to adapt to the environment as soon as possible and further improve students’ adaptability, organizations should strengthen their interventions, especially through support, such as resources, information, and funds, to promote students’ learning input and support and improve their learning performance.

#### 6.3.3. Pay attention to intervention subject organization and use authoritative channels

The effects of online teaching interventions are transmitted downward through a school’s teaching and student management groups, creating a polarization effect. This is because the intervention and communication ability of the teaching management team and the cognition among teachers and students will be more authoritative, and the intervention will also have a more positive stimulating effect. In contrast, the student management group will pay more attention to students’ lives and daily behaviors. The online teaching intervention’s strength and ability to be implemented under the student management group will be counterproductive and negatively affected, thereby reducing LPCFL. Therefore, it is better to choose more appropriate and authoritative channels for implementing online teaching interventions.

### 6.4. Limitations and future research scope

This study has some limitations. First, according to the college’s personnel training, this study focused on only foreign language teaching and students’ learning performance, specifically in one province in China. Future studies may incorporate further online-related teaching activities to better comprehend the behaviors of schools and students by conducting cross-model (online and offline) comparative research, such as comparing online teaching to offline teaching. Second, this study analyzed data from one university in China. Future researchers may collect further data from other universities in China or other countries on a larger scale to generalize the findings to China, Asia, Europe, or worldwide. Third, this study focused on a limited number of foreign language lessons. Future research may increase the lesson categories to generate more potential contributions in student settings. Fourth, owing to the complexity of the proposed model, this study did not investigate the impact of intervention activities (i.e., process, methods, tools, and environment) on students’ preferences, emotions, and knowledge acquisition. Fifth, this study has not considered the difference of intervention methods, and it is unclear whether the difference can be popularized universally or has the characteristics of online teaching. Future research could investigate this further to reveal new insights in other universities in China, across Asia, or in other countries. Finally, this study employed student input and support as mediators. Future research may apply the student learning network to uncover insightful findings on student performance.

## Data availability statement

The data analyzed in this study is subject to the following licenses/restrictions: Jiangxi University of Finance and Economics reserves the right to use the data. Requests to access these datasets should be directed to zou.yf@163.com.

## Author contributions

XY: literature review and draft. ZY: literature review, draft, data collection, and analysis. All authors contributed to the article and approved the submitted version.

## Funding

This project was funded by the High Education Teaching Reform Project of Jiangxi Province (JXJG-20-34-2) and Jiangxi Province’s “Fourteenth Five Year” Educational Science Program (22YB135 and 22YB055).

## Conflict of interest

The authors declare that the research was conducted in the absence of any commercial or financial relationships that could be construed as a potential conflict of interest.

## Publisher’s note

All claims expressed in this article are solely those of the authors and do not necessarily represent those of their affiliated organizations, or those of the publisher, the editors and the reviewers. Any product that may be evaluated in this article, or claim that may be made by its manufacturer, is not guaranteed or endorsed by the publisher.
